#  Homocystinuria: Diagnosis and Neuroimaging Findings of Iranian Pediatric patients 

**Published:** 2015

**Authors:** Parvaneh KARIMZADEH, Narjes JAFARI, MohammadReza ALAI, Sayena JABBEHDARI, Habibeh NEJAD BIGLARI

**Affiliations:** 1 Pediatric Neurology Research Center, Shahid Beheshti University of Medical Sciences, Tehran, Iran; 2 Pediatric Neurology Department, Mofid Children’s Hospital, Faculty of Medicine, Shahid Beheshti University of Medical Sciences, Tehran, Iran; 3 Department of Pediatric Endocrinology, Faculty of Medicine,Shahid Beheshti University of Medical Sciences, Tehran, Iran

**Keywords:** Homocystinuria, Neurometabolic disorder, Early detection

## Abstract

**Objective:**

Homocystinuria is a neurometabolic diseases characterized by symptoms include Neurodevelopmental delay, lens dislocation, long limbs and thrombosis.

**Materials & Methods:**

The patients who were diagnosed as homocystinuria marfaniod habits, seizure in the Neurology Department of Mofid Children’s Hospital in Tehran, Iran between 2004 and 2014 were included in our study. The disorder was confirmed by clinical andneuroimaging findings along withneurometabolic and genetic assessment fromreference laboratory in Germany. We assessed age, gender, past medical history, developmental status, clinical manifestations, and neuroimaging findings of 20 patients with homocystinuria.

**Results:**

A total of 75% of patients were offspring from consanguineous marriages. A total of 95% of patients had a history of developmental delay and 40% had developmental regression. A total of 75% had seizures from these 45% showed refractory seizures. Seizures among 13 patients werecontrolled with suitable homocystinuria treatment. The patients with homocystinuriawere followed for approximately 10 years and the follow-ups showed that the patients with an early diagnosis and treatment had more favorable clinical responses for growth index, controlled refractory seizures, neurodevelopmental status, and neuroimaging findings. Neuroimaging findings include brain atrophy and/or white matter involvement.

**Conclusion:**

According to the results of this study, we suggest that early assessment and detectionplayan important role in the prevention of disease progression and clinical signs. Homocystinuria in patients with a positive family history, developmental delays, or regression, refractory, or recurrent seizures should take precedence over other causes.

## Introduction

Homocystine is an amino acid with sulfur that comes from methionine metabolism. Homocystineis metabolized by two pathways as follows: transsulfuration or remethylation (1). Gene abnormalities in the enzymes, which catalyze the reactions in trans sulfuration or remethylation pathways, can cause hyper homocysteinuria. Homocystinuria can be caused by abnormal DNA methylation during embryogenesis(2). Hyperhomocystinuria is causedbya rare genetic error causedby deficiencies in methylenetetrahydrofolate reductase, cystathionine beta synthase, or in enzymes involved in homocystine methylation andmethyl-B12 synthesis(3). There is an association between mutations of elevated levels of homocysteine, methylenetetrahydrofolate reductase, MTHFR C677T, and increased risk of thrombosis among homozygous carriers. Heterozygote carriers for the above gene mutations with other major or minor risk factors are prone to thrombosis (4).Mutation of the methylenetetrahydrofolate reductase (MTHFR) and provides the folate derivative for homocystine to methionine conversion. This mutation can cause mild hyperhomocysteinuria. The rationale (folate supplementation) can be usedto over come the genetic deficiency in cases with low levels of folate(5). High levels of homocystine in plasma are one of the risk factors for atherosclerosis (6).It has been showed that early detection and treatment can be helpful and homocystine levels can be controlled by vitamins B6, B12, cofactors needed for homocystine metabolism, and by folic acid supplements (7-9). 

In this study, we present 10 years of experience about homocystinuria fromthe Pediatric Neurology Research Center of Mofid Children’s Hospital, Tehran, Iran. We describe clinical symptoms and neuroimaging findings for20 cases with this disorder. 

## Materials & Methods

This observational study was performed on patients who were diagnosed with homocystinuria at the Neurology Department of Mofid Children’s Hospital in Tehran, Iran, from2004–2014. The diagnosis was performed based on clinical manifestations, neuroimaging findings, homocystine level assessments in Germany,and,finally,genetic study. The data collected wereage, gender, past medical history, developmental status, general appearance, and clinical and neuroimaging findings. 

Treatment consisted of betaine, carnitine, folic acid, vitamin B12, vitamin B6 supplements, a low-protein diet, and anti-convulsant drugs in cases with seizures. The children’s diet was carefully controlled. The data were analyzed by descriptive methods and no statistical testing was applied. 

Institutional ethical approval for the conduct of this study was obtained from the Pediatric Neurology Research Center of Shahid Beheshti University of Medical Sciences, Tehran, Iran. All parents signed a written consent for participation in the study. 

## Results

Twenty patients with homocystinuria who hadbeen assessed and followed over thelast 10 years were included in this study. Sixpatients,in addition tohomocysteinuria, had methylmalonicacidemia. There were 11 males and 9 females with an agerangefrom 6-months to 15-years. The earliest case was diagnosed in a neonate who presented with a sepsis-like illness and the latest case was diagnosed in a 10 year-old with seizures due to cerebral thrombosis after lens dislocation surgery. The average age of patients at final detection time was9.6 months. A total of 75% of patients were offspring from consanguineous marriages. In addition, 5 patients had a positive family history of similar disease and2 of them died without diagnosis. 

One of our patients (39 months old) died about 2years after detection.The patient had not received any treatment due to his parent’s decision. 

Four patients had a history of neonatal hospitalization because of poor feeding in patient-number 1, was diagnosed at that time, and treatment of homocystinuria was started.Patient-number 2 had continuous vomiting that was permanent until 4 months of age and homocystinuria was detected at this time.Patient-number 3 had sepsis-like illness from12 days of age but was diagnosed at 9 months of age. Patient-number 4 had a neonatal hospitalization due to hyper bilirubinemia. 

The chief complaints in patients at detection time were seizures in 11 patients and developmental delays or regression in 7. Twocases had visual loss. In the developmental assessment, 19 patients had developmental delay, 8 patients had developmental regressions with words and movements stuck more than their recognition. Fifteen patients had seizures of which 9 patients had recurrent and refractory. The types of seizures were as follows:5 patients had generalized tonic clonic seizures; 5 had tonic seizures; 3 had partial seizures; and 2 patients had infantile spasms. The body weightof4 patients was below the 3rd percentile and the height in 6 patients wasabove the 5th percentile.Three patients had microcephaly but another patient was in thenormal developmental index. Three patients had erythematous scaling lesions on their skin. One patient had alopecia and 4 patients with homocystinuria had blond hairs and light eyes. Five patients had visual loss due to lens dislocation and secondary cataracts.One patient hadstrabismus and another patient had optic atrophy. Two patients had valvular heart disease. Seven patients had central hypotonicity . Two patients indicated stroke episodes. Six patients showed long fingers and long limbs. 

The lab data showed that all patients had increased levels of homocystine in their serum (from 48–1022 with maximum normal range of 16);6 patients had anemia;and 4 patients had megaloblastic anemia. These patients also had methylmalonicacidemia. Serum amino acid was assessedwiththe HPLC method and was showed elevated levels of glutamine in all patients, as well as increases in a few nonspecific aminoacids in some of the patients. 

**Fig 1:**
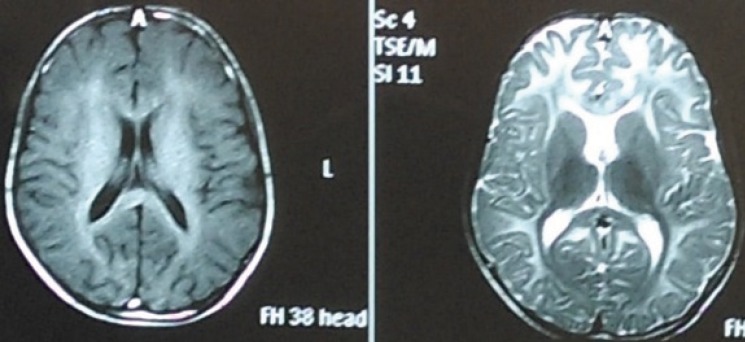
A-15-year –old male with brain involvement due to Homocystinuria

From the neuroimaging data, we saw that 11 patients had brain atrophyand white matter involvement;4 patients exhibited corpus callosum atrophy; 2 patients had only white matter involvement; 4 patients had a previous stroke; 1 patient had cerebral thrombosis; and 1patient had a normal brain MRI ( Figure 1). 

Genetic analysis was done on 3 patients and reported mutant polymorphism of C677T heterozygote and mutant allele of C homozygote. 

Patients were treated with a low protein diet, betaine, carnitine, folic acid, vitamin B12, vitamin B6, and anti-convulsant drugs in cases with seizure. Seizures were controlled in13 patients after starting anti-convulsant drugs. Regression in our patients was stopped after starting treatment with 5 out of 8 patients progressed to have the ability again.Even white matter involvement and brain atrophy improved after treatment. Also further strokes after treatment did not occur. Only one patient who was 39 months old did not receive special treatment anddied due to a refractory seizure.

## Discussion

Homocystinuria is a rare inborn error of the metabolismwith autosomal recessive inheritance that is caused byenzyme deficiencies(3). Homocystinuria is caused by genetic mutations in the enzymes and may contribute to increases in plasma homocystine. First, Carson and Neill reported the association between mental retardationand homocystinuria in two Irish brothers in 1962 (10). In 1964, Gerritsen and Waisman first identified homocystine in the urine and defined homocystinuria (11). Prior to the 1960s, in 1933, homocystinuria had already been described in an eight-year-old child with mental retardation, dislocation of the lens, and skeletal abnormalities with coxavara and who died from a stroke (12). Homocystinuria was diagnosed in this child’s nephew in 1965 (13). Normal levels of plasma homocysteine are between 5–15 nmol/ml and concentrations between 16–30 nmol/ml is mild, 31- 100nmol/ml is moderate, and greater than100 nmol/ ml is severe hyperhomocystinuria(14). MuddSHet al reported that around half of their cases had a good laboratory and clinical response to high doses of vitamin B6 (15). Fonseca et al had no known relevant clinical findings in family members (16). Treatment for elevated homocystine levels is simple and innocuous. High doses of pyridoxine (B6) were used initially with success in children with homocystinuria (17). 

The clinical features include subluxation of the lens, which are characteristic for connective tissue disorders (15). Our results were similar and five patients had visual loss from lens dislocation and secondary cataracts. Twenty patients (11 males and 9 females) with homocystinuria were included in this study. A total of 75% of cases were fromconsanguineous parents and 25% had similar diseases in their families. Therefore, in suspected cases of homocysteinuria, having consanguineous parents can contribute to the diagnosis because of autosomal recessive inheritance of homocysteinuria. A total of 15% of patients had a history of hospitalization due to period of sepsis-like illness that metabolic disease assessment can be helpful indetection forill neonates. A total of 95% of patients had a history of developmental delay and 40% had developmental regression. Patients with these symptoms who are referred to a pediatric neurologist, it is our suggestion to for the pediatric neurologist to consider homocysteinuria. A total of 35% of patients had heights greater than normal and 40% of patients had skin and hair involvement, i.e. skin lesions and blond hair, among others. 

From brain MRIs, 75% of patients had brain involvement (generalized atrophy, white matter involvement, previous infarct pattern, and venous sinus thrombosis). In follow-up imaging, which was done in 6 patients, 50% of them had improvements in white matter involvement and brain atrophy. Sachdeva Vet al reported that all patients had ischemic lesions in the brain MRI with contrast (18). 

A total of 75% of patients had seizures and 45% of these seizures werere current and refractory. Seizures were controlled for13 patients after starting anti-epileptic drugs and special treatment for homocysteinuria. Regression in our patients was stopped after starting treatment and 62% of patients regained that ability.In addition, further strokes after treatment did not occur. 

Our patients with homocystinuria came to our specialist center and exact evaluationswere done. 

In conclusion, based on our results, patients with developmental delay or regression, long limbs and tall, recurrent seizures, skin lesions, blond hair, eye involvement such as secondary cataracts due to lens dislocation, hypotonia, brain involvement in MRI include brain atrophy or white matter involvement, and having a positive family history of homocystinuria thenhomocystinuria disorder should be considered. 

## Notes

**Declaration of conflicting interests: **None declared.

## Notes

**Funding: **The authors received no financial support for the research and publication of this article.
